# Event-Based Stereo Depth Estimation Using Belief Propagation

**DOI:** 10.3389/fnins.2017.00535

**Published:** 2017-10-05

**Authors:** Zhen Xie, Shengyong Chen, Garrick Orchard

**Affiliations:** ^1^College of Computer Science, Zhejiang University of Technology, Hangzhou, China; ^2^Temasek Laboratories, National University of Singapore, Singapore, Singapore; ^3^Singapore Institute for Neurotechnology (SINAPSE), National University of Singapore, Singapore, Singapore

**Keywords:** event-based camera, stereo matching, event-driven, message passing, belief propagation, disparity map

## Abstract

Compared to standard frame-based cameras, biologically-inspired event-based sensors capture visual information with low latency and minimal redundancy. These event-based sensors are also far less prone to motion blur than traditional cameras, and still operate effectively in high dynamic range scenes. However, classical framed-based algorithms are not typically suitable for these event-based data and new processing algorithms are required. This paper focuses on the problem of depth estimation from a stereo pair of event-based sensors. A fully event-based stereo depth estimation algorithm which relies on message passing is proposed. The algorithm not only considers the properties of a single event but also uses a Markov Random Field (MRF) to consider the constraints between the nearby events, such as disparity uniqueness and depth continuity. The method is tested on five different scenes and compared to other state-of-art event-based stereo matching methods. The results show that the method detects more stereo matches than other methods, with each match having a higher accuracy. The method can operate in an event-driven manner where depths are reported for individual events as they are received, or the network can be queried at any time to generate a sparse depth frame which represents the current state of the network.

## 1. Introduction

Traditional frame-based stereo vision systems continue to steadily mature, in part thanks to publicly available datasets, such as the Middlebury (Scharstein and Szeliski, 2002) and KITTI (Menze and Geiger, 2015) benchmarks. Recently, new frame-based hardware stereo devices have entered the commercial market such as the ZED, VI-sensor, and Realsense. Despite advances in algorithms and hardware, frame-based stereo algorithms still struggle under certain conditions, especially under rapid motion or challenging lighting conditions.

Even under ideal conditions, the latency of frame-based stereo vision sensors is typically on the order of 50–200 ms, including the time required for both data capturing and processing. Latency can be reduced and accuracy improved through brute force use of higher frame rate cameras and more powerful computing hardware. However, for applications where high speed stereo sensing is required, such as indoor flight with a small aerial vehicle, the Size, Weight, and Power (SWAP) available for sensing and computing is severely limited.

Event-based vision sensors loosely mimic biological retinas, asynchronously generating events in response to relative light intensity changes rather than absolute image intensity (Posch et al., 2011). Event-based vision sensors have desirable properties for operating in uncontrolled environments. They provide sparse data with little redundancy at low latency and high temporal resolution over a wide intra-scene dynamic range. These properties are beneficial for sensing from a mobile vehicle where computing resources are limited, but low latency sensing is still required, and the lighting of the surroundings cannot be controlled. However, the traditional frame-based algorithms are not well suited to operate on event-based data. In this paper, we present a fully event-based stereo matching algorithm for reliable 3D depth estimation using a method based on message passing.

The stereo matching problem for estimating depth from 2D images plays an important role in sensing for mobile vehicles. Frame-based stereo matching methods can be categorized into active and passive approaches. The Kinect and Realsense are active sensors (they emit a structured light pattern in infrared). These active methods are remarkable for their real-time and stable performance. However, they suffer from limited range since IR strength falls off with distance, and they struggle in the presence of ambient IR light, especially direct sunlight.

Passive methods directly process pairs of images, as is done by the ZED and VI-sensor. These methods usually have relative long detection range and high resolution depth map but require visual features for matching and typically require a powerful CPU or GPU to process in real-time. Event-based stereo matching works by finding corresponding events from two different views and estimating the disparity. Event-based stereo is a passive approach since there is no emission. The events used to consist of only a time, polarity (direction of change), and pixel address. They do not directly encode absolute intensity which is typically used in frame-based stereo matching.

Many researchers have explored event-based matching criterions for event-based cameras such as ATIS (Posch et al., 2011) and DVS (Lichtsteiner et al., 2008). Kogler et al. (2011) focused on using the temporal and polarity correlation to match different events and achieved promising initial results. However, matching using temporal and polarity criterion alone is prone to errors because the latency of events varies (jitter) (Rogister et al., 2012). This problem is more obvious when multiple objects are moving in the field of view. Rogister et al. considered not only timing constraints, but also geometry constraints, event ordering, and temporal activity constraint. Their method still got relatively low reconstruction accuracy because ambiguities cannot be uniquely solved from these temporal and geometrical constraints alone (Camuñasmesa et al., 2014).

Benosman et al. have several papers on stereo (Camuñasmesa et al., 2014; Lagorce et al., 2017) which explore the use of spatial, temporal, orientation, and motion constraints such as orientation and Time-Surfaces for event-based stereo matching.

These methods usually have low estimation rate (ratio of depth estimates to input events) and still produce many false stereo matches. These event-based algorithms do not consider the 3D constraints between adjacent events in the physical world. However, benchmarks for frame-based algorithms show that state-of-art global or semi-global frame-based stereo matching methods consider both disparity uniqueness constraints and disparity continuity constraints. These constraints can also be applied to event-based stereo (Besse and Rother, 2014).

Firouzi and Conradt (2016) came up with the dynamic cooperative neural network to make use of the stream of the events. The method used the idea from Marr's cooperative computing approach (Marr, 1982) but made it dynamic to take into account the temporal aspects of the stereo-events. The result shows that the estimation rate considerably outperformed previous works. However, the algorithm is sensitive to scene dependent parameters which cannot necessarily be estimated beforehand, as we show later in section 2.3.

Inspired by Cook et al. (2011) who was using message passing algorithm to jointly estimate ego-motion intensity and optical flow, we explore message passing for stereo depth estimation.

The main idea of our algorithm is borrowed from message-passing algorithms. Message passing algorithms are used to solve inference, optimization, and constraint satisfaction problems. For these problems, the inputs are noisy or ambiguous measurements within a specific model and the output is the most probable state of some hidden state or attributes (MacKay, 2003).

In this work, we use Belief Propagation (BP), which is also known as sum-product or max-product message passing. BP is a message passing algorithm for performing inference on graphical models, such as Bayesian networks and Markov random fields (MRF). BP calculates the marginal distribution for each unobserved node, conditional on any observed nodes (Koller and Friedman, 2009).

**Figure 1:**
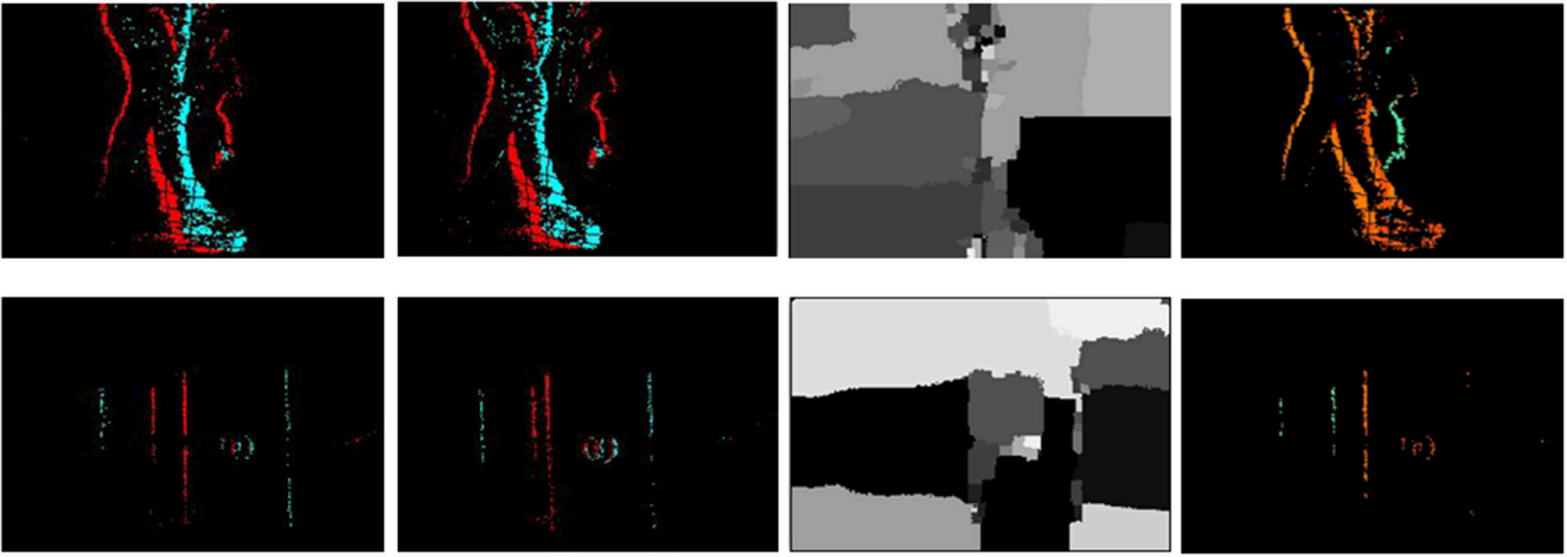
Results from the classical BP and the proposed algorithm. The upper row is the recording of two people walking generated by accumulating 10 ms of events. The left to right are the left and right input (color means polarity), the result of frame-based BP (grayscale value means disparity) and the result of our proposed algorithm (color means disparity). The lower row shows the recording of two boxes.

Stereo matching can be defined as a labeling problem. The labels correspond to the disparity. Generally, the quality of labeling is defined as a cost function. Finding labels that minimize the cost corresponds to a maximum a posteriori (MAP) estimation problem in an appropriately defined MRF (Sun et al., 2003 Felzenszwalb and Huttenlocher, 2004). BP as a global cost optimization method is used by some state-of-art frame-based stereo methods on the Middlebury and KITTI benchmarks. However, traditional BP algorithms can only operate on static features to solve the correspondence problem. It does not consider continuity between the current frame and next frame. In this work, our input data is a stream of events instead of pairs of images. One possible method for estimating stereo is to construct frames by accumulating events over a period of time, and then use the BP method (Felzenszwalb and Huttenlocher, 2004) to process in a frame-based manner. However, as shown in Figure 1, the classical BP does not work for event accumulated frames, so we have to construct a modified MRF to manage the event-driven input and formulate a dynamic updating mechanism to deal with the temporal correlation of the event stream. Although events have no persistent measurement of intensity which is the key feature used in frame-based stereo methods, the constraints such as local smoothness consistency and disparity uniqueness are still valid and useful in event-based stereo matching.

The major contributions of our method are as follows:

1. Proposing a method for using message passing to solve the event-based stereo matching problem.
2. Exploring increasing the density of depth estimates from the event-based sensor.
3. Evaluating our method and others on recordings which include ground truth obtained from the ZED sensor.

After validating our method compared with several state-of-art event-based stereo matching methods on our datasets, the results demonstrate our method has a higher estimation rate and estimation accuracy. In other words, it produces more depth estimates, and with higher depth accuracy. Additionally, our methods have the advantage of providing a slightly more dense depth map. An illustrative video of our algorithm^1^ and the data and source code^2^ can be found online.

## 2. Materials and methods

In this section we first describe the hardware setup for the stereo rig (section 2.1), before describing our algorithm (section 2.2), and how the algorithm was tested (section 2.3).

### 2.1. Hardware setup

**Figure 2:**
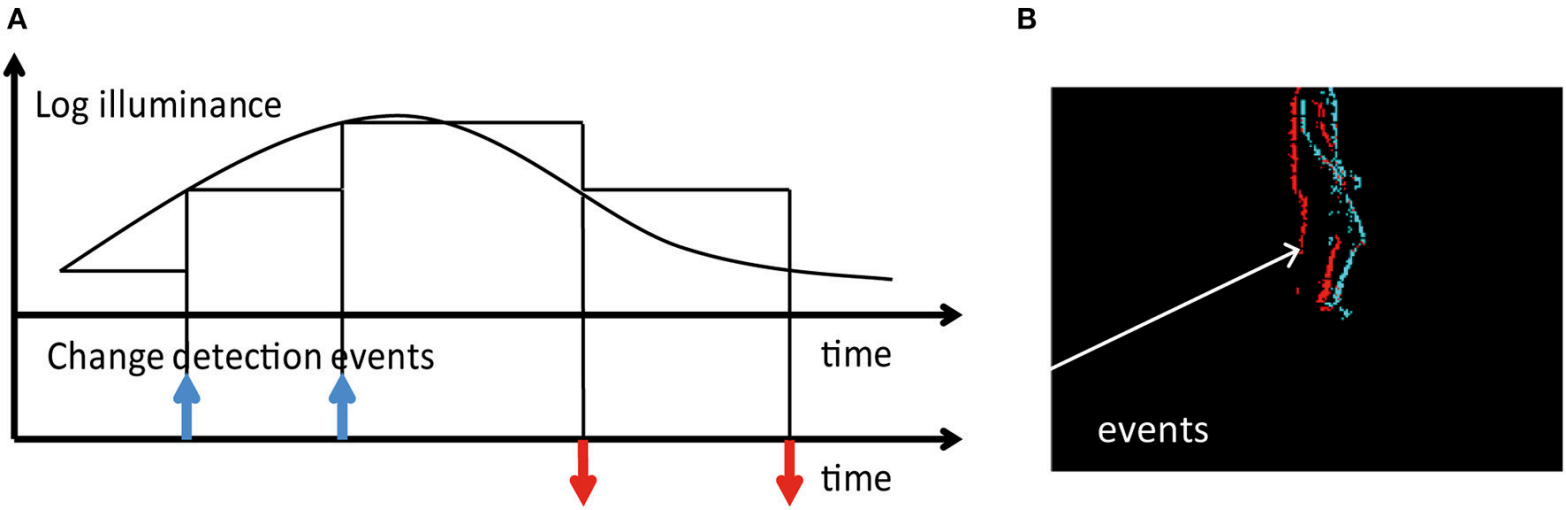
**(A)** Principle of operation for DAVIS pixels. Each pixel produces an event whenever its log-illumination changes by a fixed amount. ON and OFF events are generated by increases and decreases in intensity respectively. **(B)** An image generated by accumulating 20 ms of events. Cyan and red pixels indicate the locations of ON and OFF events respectively.

For the event-based stereo setup, we rely on two DAVIS240C (Brandli et al., 2014) sensors. The DAVIS family of sensors combine asynchronous event-based temporal contrast detection with a synchronous frame-based readout. Such a setup allows for the capture of both intensity frames and temporal contrast events. In this work, we rely on the events only. Temporal contrast events are generated by pixels independently and asynchronously as and when changes in intensity occur in the scene. Figure 2 shows how events are generated.

**Figure 3:**
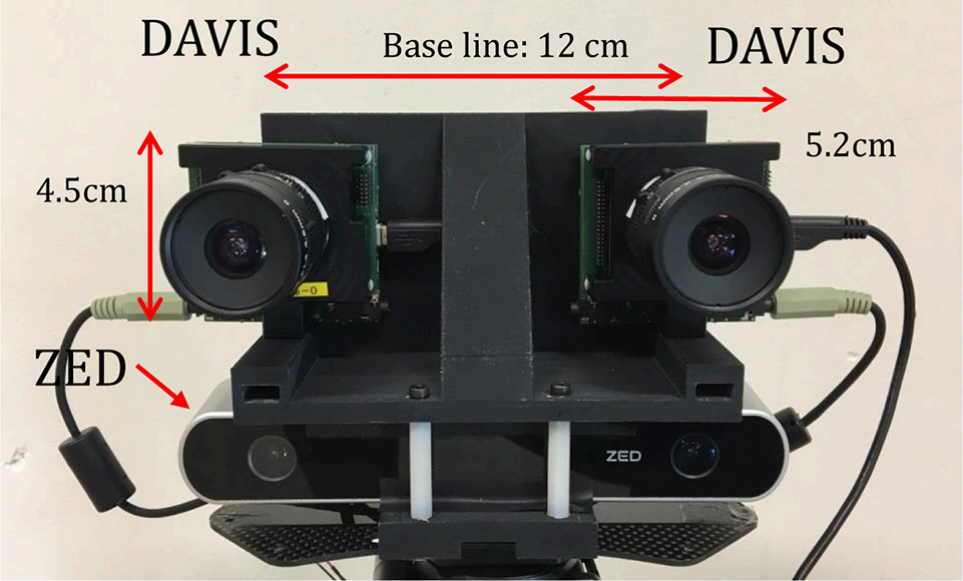
The stereo camera setup consisting of two DAVIS240C sensors (top) mounted above a ZED sensor using a 3D printed plastic mount. The entire setup is mounted on a tripod and calibration is used to accurately determine the pose of all four cameras.

Figure 3 shows the stereo rig used to capture data and evaluate algorithm performance. It consists of the ZED frame-based stereo sensor mounted below two event-based DAVIS240C sensors, all of which are held together with a 3D printed plastic mounting.

Events are read out from each DAVIS240C sensor independently over two separate USB cables, but their timestamps are synchronized using the standard timestamp synchronization feature of the sensors (which relies on the audio cable seen in the figure).

The designed baseline of the event-based cameras is 12 cm. The KOWA F1.4, 4.5 mm lenses provided with the sensors were used. Based on the parameters of the cameras and lens, the best possible depth detection range is from 0.6 m (50 pixel disparity) to 30 m (1 pixel disparity), assuming that disparity can only be calculated in steps of 1 pixel (with frame-based methods sub-pixel accuracy is known to be possible).

The ZED sensor is used to generate an approximation of ground truth for comparison. ZED is capable of a resolution of 672 × 376 pixels at 100 Hz. However, in practice, the maximum frame rate used for ZED recordings is limited by the IO speed of hard disk. The ZED sensor records the SVO file (StereoLabs video file format) containing additional ZED data other than the images. Data from the DAVIS240C, ZED, and Vicon are simultaneously recorded using the Robot Operating System (ROS)^3^.

In order to register events against ZED depth estimates, the ZED sensor is calibrated against the left DAVIS sensor to get the precise relative position. Then we use a similar approach to Weikersdorfer et al. (2014), which uses the smallest depth value within a one-pixel neighborhood in the most recent frame.

### 2.2. Algorithm

**Figure 4:**
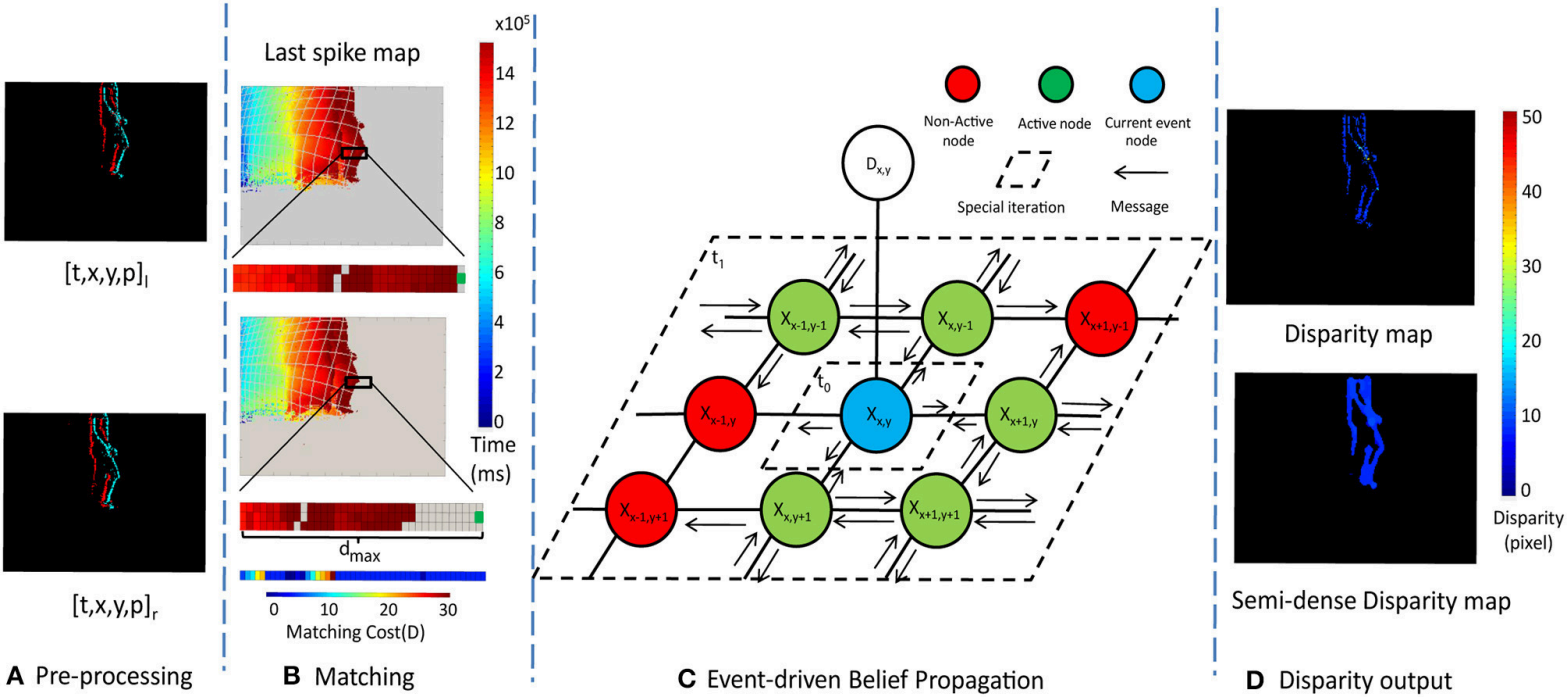
The framework of the message passing algorithm. **(A)** is the Pre-processing (section 2.2.1), **(B)** is the Event stream Matching (section 2.2.2), **(C)** is Event-driven Belief Propagation (section 2.2.3) and **(D)** is Disparity output (section 2.2.4)

Figure 4 shows the outline of the stereo algorithm which consists of four main steps: Pre-processing, Event-based Stereo Matching, Event-driven Belief Propagation, and Disparity output. Each of these steps will be described in a separate subsection below.

The input to the algorithm is a stream of events, where the *i*th event can be represented as a vector *e*_*i*_ = [*c*_*i*_, *t*_*i*_, *x*_*i*_, *y*_*i*_, *p*_*i*_], where *c*_*i*_ indicates which camera the event came from, *t*_*i*_ is the time of the event, [*x*_*i*_, *y*_*i*_] is the pixel location of the event, and *p*_*i*_ is the polarity, indicating whether the event was caused by an increase or decrease in intensity. The output of the stereo algorithm is also a stream of events, *E* = (*t*_*i*_, *x*_*i*_, *y*_*i*_, *p*_*i*_, *d*_*i*_), where *d*_*i*_ is the disparity in pixels.

The *pre-processing* stage performs noise filtering and stereo rectification. The rectified events are passed to the *event-based stereo matching* stage which identifies potential matches between rectified events from the two sensors. These potential matches are passed to the *event-driven belief propagation* step which enforces disparity smoothness and uniqueness constraints to choose between multiple potential stereo matches. Finally, the *disparity output* stage estimates the disparity of each event and generates a semi-dense disparity map with the updated MRF. By *semi-dense* we mean the output is not sparse event points but more dense structures such as edges and boundaries.

#### 2.2.1. Pre-processing

The pre-processing stage consists of noise filtering and rectification. First, the input event streams from each of the two sensors are individually noise filtered. A simple nearest neighbor filter was used, which filters out an event if no neighboring pixels generated an event in the preceding 30 ms (Czech and Orchard, 2016). Second, rectification is performed.

Given a pair of stereo images, rectification determines a transformation of each image, such that the resulting transformed images appear as if they were captured by two coplanar image sensors aligned such that each row of pixels in the left sensor lies on the same line as the corresponding row of pixels in the right sensor. The important advantage of rectification is that computing stereo correspondences is reduced to a 1-D search problem along the horizontal raster lines of the rectified images (Fusiello et al., 2000) rather than a full 2D search. The first step in rectification is to calibrate the sensors. Calibration is performed by using the frame-capture capability of the DAVIS240C to simultaneously record frames from both sensors, which can then be used with OpenCV to calibrate. Simultaneous calibration of both sensors also provides the parameters required for rectification.

For each event's pixel location, the corresponding location in the rectified image can be computed. However, this location will typically lie somewhere between integer pixel locations. In this case, we round off the pixel location to the nearest integer value. This rectification maps [*x*_*i*_, *y*_*i*_] pixel locations of the original events to modified pixel locations $\left[x_{i}^{\prime },y_{i}^{\prime }\right]$ in the rectified events. Forcing $\left[x_{i}^{\prime },y_{i}^{\prime }\right]$ to be integer values may cause some loss in accuracy, but it simplifies the algorithm by preventing the need to keep track of sub-pixel locations.

**Figure 5:**
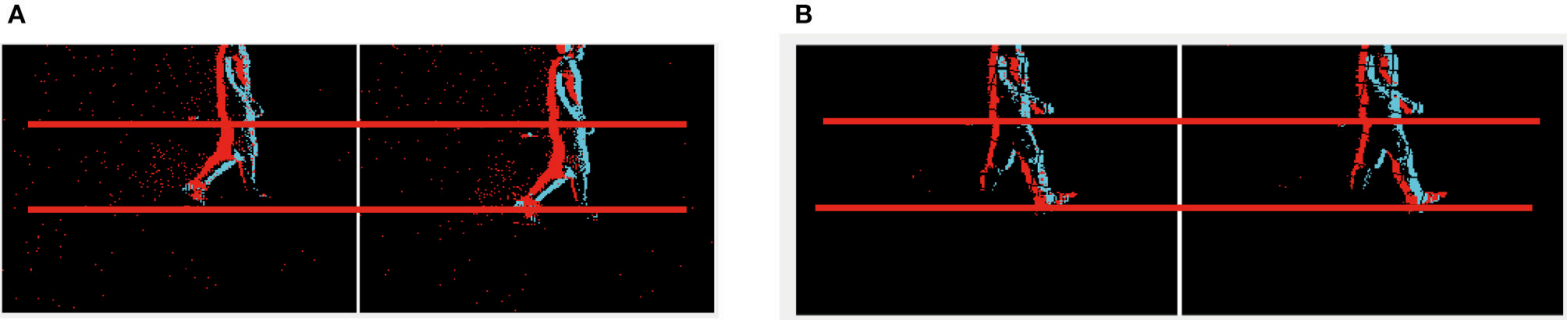
**(A)** The snapshot of the raw events. **(B)** The snapshot of the rectified events.

Since the mapping is one-to-one, there is no need to recompute the rectification transformation and find the nearest pixel location for each event. Instead, we compute the transformation once at startup and populate a lookup table to speed up the computation. The results from rectification are shown in Figure 5.

#### 2.2.2. Event-driven matching

After pre-processing, an input event from the left camera, $e_{i}^{\prime }=\left(0,t_{i},x_{i}^{\prime },y_{i}^{\prime },p_{i}\right)$ will have potential matching events in right camera $e_{j}^{\prime }=\left(1,t_{j},x_{j}^{\prime },y_{j}^{\prime },p_{j}\right)$ which match the criteria:

(1) $$\begin{array}{l} |t_{j}^{\prime }-t_{i}^{\prime }|\;\leq \tau_{t},\\ |y_{j}^{\prime }-y_{i}^{\prime }|\;\leq 1,\\ 0\leq x_{i}^{\prime }-x_{j}^{\prime }\leq d_{max},\\ p_{i}\;=p_{j}, \end{array}$$

where *d*_*max*_ is the maximum disparity. In other words, for two events to be considered a possible stereo matching pair, they must be from different sensors, must occur within τ_*t*_ milliseconds of each other, must have the same polarity, must be from the same or neighboring rows (*y*). The *x*-address of the left sensor event must be greater than or equal to the *x*-address of the right sensor event, but not by more than *d*_*max*_ pixels.

For implementation, we keep track of the time of the last spike of each polarity from each sensor. We have a memory array of size *W* × *H* × 2 × 2, where *W* and *H* are the width and height of the sensor in pixels, and there are two polarities and two sensors. Each location in the memory array holds the time of the last spike for the corresponding sensor, pixel location, and polarity.

An example of the contents of this memory array generated by a single person walking across the scene is shown in Figure 4B. Gray areas indicate where no event has occurred since the beginning of the recording, while other colors indicate when the most recent event for each pixel occurred.

For each incoming event, *e*_*i*_, of the left camera, the candidate region for events in the right camera which match the criteria given in Equation (1) can be extracted from the last spike map, and the cost for these candidate matches at each disparity, *d*, can be computed using:

(2) $$\begin{array}{l} C_{t}(d,y_{j})=\frac{\left|t_{i}-t_{j}\right|}{\epsilon_{t}}\\ C_{g}(d,y_{j})=\frac{\left|y_{i}-y_{j}\right|}{\epsilon_{g}}\\ C_{total}(d,y_{j})=C_{t}(d,y_{j})+C_{g}(d,y_{j})\\ D(d)=\{\begin{array}{ll} \min_{y_{j}}(C_{total}(d,y_{j})), & \text{if }\min_{y_{j}}(C_{total}(d,y_{j}))<D_{max}\\ C_{max}, & \text{otherwise} \end{array} \end{array}$$

were *C*_*t*_(*d, y*_*j*_) is a cost term which penalizes potential matches proportionally to the event time differences, *C*_*g*_(*d, y*_*j*_) is a cost term which penalizes potential matches proportionally to how far they lie from the epipolar line. *D*(*d*) is the total matching cost for disparity *d*, known as the data term. It is chosen as the minimum of the costs for any potential matches at disparity *d*, where *d* ranges from 0 to *d*_*max*_. *D*_*max*_ is a saturation term used to limit the maximum value of the data term.

#### 2.2.3. Event-driven belief propagation

For the event-driven message passing framework, we follow the idea from Felzenszwalb and Huttenlocher (2004), which defines stereo matching as a labeling problem. Let *P* be the set of pixels of the image and *L* be a set of labels corresponding to the disparity. A labeling *d* assigns a label *d*_*p*_ ∈ *L* to each pixel *p* ∈ *P*. The quality of labeling is given by a cost function:

(3) $$E(d)=\sum_{p\in P}(D(d_{p})+\sum_{q\in N(p)}V(dp,dq)),$$

where *N*(*p*) is the neighborhood of pixels around *p*. *D*(*d*_*p*_) is the data term which represents the cost of assigning disparity *d*_*p*_ to pixel *p*, calculated from Equation (2). *V*(*dp, dq*) is the cost of assigning labels *d*_*p*_ and *d*_*q*_ to two neighboring pixels (defined later) and is referred as discontinuity cost, which enforces spatial smoothness of the disparity. Our goal is to find proper label for each pixel to minimize the cost, which corresponds to a maximum a posteriori estimation problem in an appropriately defined Markov Random Field(MAP-MRF). The max-product BP algorithm can be used to solve the MAP-MRF problem efficiently (Felzenszwalb and Huttenlocher, 2004).

A schematic of the MRF connectivity and messages are shown in Figure 4C. The graph consists of a hidden node, *X*_*x, y*_, for each pixel location [*x, y*]. Each hidden node is connected to its four nearest neighbors, and an observation node, *D*_*x, y*_.

The state of the observation node is *D*_*x, y*_ = *D*(*d*) from Equation (2), and is used to calculate the hidden state *X*_*x, y*_ as well as the messages traveling from the hidden node to its neighbors.

The hidden node state presents the posterior probability distribution over possible discrete disparities. The negative log of the probabilities is used to make the max-product become a min-sum, which is less sensitive to numerical artifacts and directly corresponds to the cost function definition Equation (3).

The state of each hidden node is presented as a *d*_*max*_ dimension vector. Each dimension stores the cost of a certain disparity. The cost is determined by a combination of the observation data, and the messages from the neighboring nodes.

Traditionally the max-product BP algorithm works by simultaneously passing messages around the whole graph defined by the four-connected image grid.

Our algorithm does not simultaneously update the entire graph. Rather, whenever a new observation is available from the stereo matching step, only the neighborhood of the observation will be updated.

Each message is also a *d*_*max*_ dimension vector. We use the notation $m_{p\to q}^{t}\left(d_{q}\right)$ to denote a message that node *p* sends to neighboring node *q* at iteration *t*. At algorithm initialization, all message values $m_{p\to q}^{0}\left(d_{q}\right)$ are initialized to zero. Thereafter, messages are calculated as follows:

(4) $$\begin{array}{l} m_{p\to q}^{t}(d_{q})=H(\tau_{m}-\Delta t)\underset{d_{p}}{\min}[V(d_{p},d_{q})+D_{p}(d_{p})\\ \;+\sum_{s\in N(p)\backslash q}m_{s\to p}^{t-}(d_{p})]\\ V(d_{p},d_{q})=\frac{\left|d_{p}-d_{q}\right|}{\epsilon_{d}} \end{array}$$

where *V*(*d*_*p*_, *d*_*q*_) is the degree of difference between the neighboring labels. In this case, the difference of the disparity is used. *N*(*p*)\*q* denotes the neighbors of *p* other than *q*. $m_{s\to p}^{t-}$ means the previous message value update by the iteration at the current time or by previous node updating. *H*()^·^ is the Heaviside step function, ensuring that only nodes active within the last τ_*m*_ seconds are considered (Δ*t* is the time since the last update for the node). Only active nodes are used to update the messages. Inactive nodes do not generate messages, they only receive messages. We the min convolution algorithm from Felzenszwalb and Huttenlocher (2004) to reduce the complexity of message updating to be linear rather than quadratic in the number of labels.

Using Figure 4C as an example, when a new event is processed, its matching cost computed using Equation (2) and used to update the corresponding observation node *D*_*x, y*_. For the first iteration *t*_0_, the hidden node *X*_*x, y*_ calculates and passes the messages to its neighbor nodes using Equation (5). Then for the second iteration *t*_1_, each nodes in the neighborhood calculate messages using Equation (5) for its own four-connected neighborhood. The spatial regions within which messages are updated in steps *t*_0_ and *t*_1_ are shown in Figure 4.

After the message iteration process has completed, the belief vector at each node is computed as:

(5) $$b_{p}(d_{p})=D_{p}(d_{p})+\sum_{s\in N(p)}m_{s\to p}^{T-}(d_{p})$$

where *b*_*p*_(*d*_*p*_) is the belief for node *p*.

#### 2.2.4. Disparity output

The steps described thus far generate a belief vector for each node. For Max-Product Belief Propagation, the goal is to find a labeling with maximum posterior probability, or equivalently with minimum cost. We select the label *d*_*p*_ which minimizes *b*_*p*_(*d*_*p*_) as the best disparity for the node. If the cost of the best disparity, *b*_*p*_(*d*_*p*_), is greater than an outlier threshold τ_*o*_ then no disparity output is generated for the node.

There are two methods for getting disparity output from the network. For the first method, whenever there is a new observation (caused by the arrival of a new input event), the most likely disparity at the location of the observation can be output. This method is event-driven because output disparities are directly driven by input events. For the second method, beliefs and disparities can be calculated from the network state for all locations whenever a disparity map is requested. For the sake of visualization, this is typically done at constant time intervals (frame intervals).

The BP method can assign disparities to nodes for which no observations are available, which results in slightly more dense depth estimates than would be achieved by simply matching events. However, the local nature of the message passing updates means we only estimate disparities for pixels within a small region around data observations.

#### 2.2.5. Overview

The general workflow of the algorithm is depicted in Algorithm 1.

**Algorithm 1:** Event-driven Message passing Stereo Matching

Initialize last spike time map, MRF and the parameters
**for** each incoming event, *e* = (*c, t, x, y, p*) **do**
Update last spike time map
Construct set of possible corresponding candidates, using Equation (1)
**for** each candidate matching pair *C*_*e*_, *d*_*k*_|0 ≤ *d*_*k*_ ≤ *d*_*max*_ **do**
Calculate temporal and geometrical difference as data cost term using Equation (2)
**end for**
Update messages locally around x,y using Equation (5)
Compute belief in x,y using Equation (6)
Select disparity which minimizes cost (if less than outlier threshold τ_*o*_).
Store the time at which each node was updated for future use in Equation (5)
**end for**

### 2.3. Experiment setup

Our testing investigates three main areas. The first set of tests compares our algorithm against three other event-based stereo matching algorithms. The second set of tests presents the semi-dense output of our algorithm. The third tests show a brief comparison of frame-based and event-based stereo in a scene with challenging lighting conditions.

There are some state-of-art event-based stereo matching algorithms like Rogister's (Rogister et al., 2012), Camuñasmesa (Camuñasmesa et al., 2014), and Firouzi's (Firouzi and Conradt, 2016). Rogister et al. used one moving pen and two simultaneously moving pens as stimulus and showed the detected disparity (Rogister et al., 2012), but the accuracy of the algorithm is not quantitatively analyzed. Camuñasmesa et al. also used simple objects like ring, pen, and cube to do the evaluation and reported the estimation rate and correctly matched events. But the correctly matched events are estimated by subtracting the isolated and incorrectly matched events from the total number of matched events (Camuñasmesa et al., 2014). There is no ground truth of depth for each event to precisely analyze the results.

Firouzi et al. used more complex stimulus such as hands shaking in different depth. However, the ground truth is estimated by manually measuring the distance between the camera and object and assumed all the triggered events are in the same disparity. Recently, some datasets for event-based simultaneous localization and mapping (SLAM) (Kogler et al., 2013; Serrano-Gotarredona et al., 2013) have become available, but none of those are created for event based stereo matching and the above previous works do not release their test datasets.

In this paper we have replicated Rogister's and Firouzi's algorithms. For Luis's algorithm, orientation matching requires the sensors to have the same orientation, and is therefore not a very general method. Orientation estimation could instead be done after rectification, but in our case, rectification leaves gaps in the images (some rectified pixel locations do not map to any pixels in the original scene). To extract orientations from rectified data with holes, the size of the filters would need to be increased, thereby reducing location specificity, and making them a poor choice for stereo matching. We nevertheless combined other constraints mentioned in Luis's work with a novel restriction Timesurface (Lagorce et al., 2017) as a comparison. Meanwhile, we collect our own datasets including not only simple rigid object such as the boxes but also flexible object like walking people with depth ground truth. Besides, the datasets also include stereo events, grayscale images, depth and camera motion. The datasets can be used not only for stereo but also for scene flow, SLAM, and other event-based applications.

### 2.4. Data

The datasets used in previous works (Rogister et al., 2012; Camuñasmesa et al., 2014; Firouzi and Conradt, 2016) both assume the cameras are static. For the comparisons with previous algorithms, we also use recordings from a static stereo rig. We use five recordings for comparison. The recordings are listed below.

1. One box moving sidewise (*One box*).
2. Two boxes at different depths moving sidewise (*Two boxes*).
3. One person walking sidewise (*One person*).
4. Two people in different depth walking sidewise (*Two people*).
5. One person walking from near to far (*One person different depth*).

To select parameters to use in the comparison, we started with parameters from the previous algorithms and then fine tuned them by hand on the *one box* recording (most similar to the datesets of previous work) to achieve the best result. The same parameters were then used for the other four recordings. The main parameters of our algorithm are set as follows: τ_*t*_ = 20 ms , τ_*m*_ = 10 ms , *d*_*max*_ = 50, ϵ_*t*_ = 3 ms , ϵ_*g*_ = 3, ϵ_*d*_ = 1, *D*_*t*_ = 5, τ_*o*_ = 1. τ_*t*_ used in Equation (1) and τ_*m*_ used in Equation (5) are the temporal outlier threshold for matching and belief propagation, which is set according to the expected object speed (τ_*t*_ is usually from 10–30 ms while τ_*m*_ is half of the τ_*t*_). *d*_*max*_ used in Equation (1) decides the maximum possible disparity (lowest possible depth). *d*_*max*_ = 50 means the possible depth detection range is from 0.6 m. ϵ_*t*_ and ϵ_*g*_ used in Equation (2) give the weight of temporal and spatial cost (ϵ_*t*_ is usually from 1ms to 3 ms while ϵ_*g*_ is from 1 to 3). τ_*o*_ is the outlier threshold. Higher τ_*o*_ increases the estimation accuracy but decreases the estimation rate.

To evaluate the performance of each algorithm, the disparity map and the disparity histogram are used. The disparity maps are accumulated with 40 ms events with each pixel representing an event and the color map of jet presenting the disparity. The blue pixel color corresponds to a disparity close to 0 while the red color corresponds to a disparity close to 50. The disparity histograms are created to show the number of the events with a certain disparity.

In order to quantitatively evaluate the result, we use three measures of accuracy. The first measure is *estimation rate*, which is the ratio of stereo matches detected divided by the number of input events from the left camera.

The second measure is *estimation accuracy*, which is the percentage of estimated disparities which are within 1 pixel disparity of the ground truth (obtained from ZED and the event-based camera and ZED were calibrated against each other) (Kogler et al., 2011). The third measure is the *depth accuracy*, measured as a percentage, defined as:

(6) $$z_{acc}(\Theta )=\frac{\sum_{i\;=\;1}^{N}H(\Theta -|\frac{{z^{\prime }}_{i}-z_{i}}{z_{i}}|)}{N}\;$$

where *z* indicates depth (z-direction from the camera), $z_{i}^{\prime }$ and *z*_*i*_ are the estimated and ground truth depths respectively. Θ is the error tolerance percentage, *H*(·) is the Heaviside step function, and there are *N* depth estimates generated for the sequence. *z*_*acc*_(Θ) gives the percentage of estimates for which the error is below the threshold of Θ%.

In the experiments, *ST* is used to denote Rogister's method which enforces Space (epipolar) and Time constraints for stereo matching. *STS* is used to denote matching based on Spatio-Temporal Surfaces (Lagorce et al., 2017). *Cop-net* is used to denote Firouzi's cooperative network approach, and *EMP* is used to denote our Event-based Message Passing approach. All the algorithms are implemented in MATLAB2015b 64-bit, running on an Intel I7 3.4 Ghz processor with 32GB RAM.

## 3. Results

### 3.1. Estimation rate and accuracy

**Figure 6:**
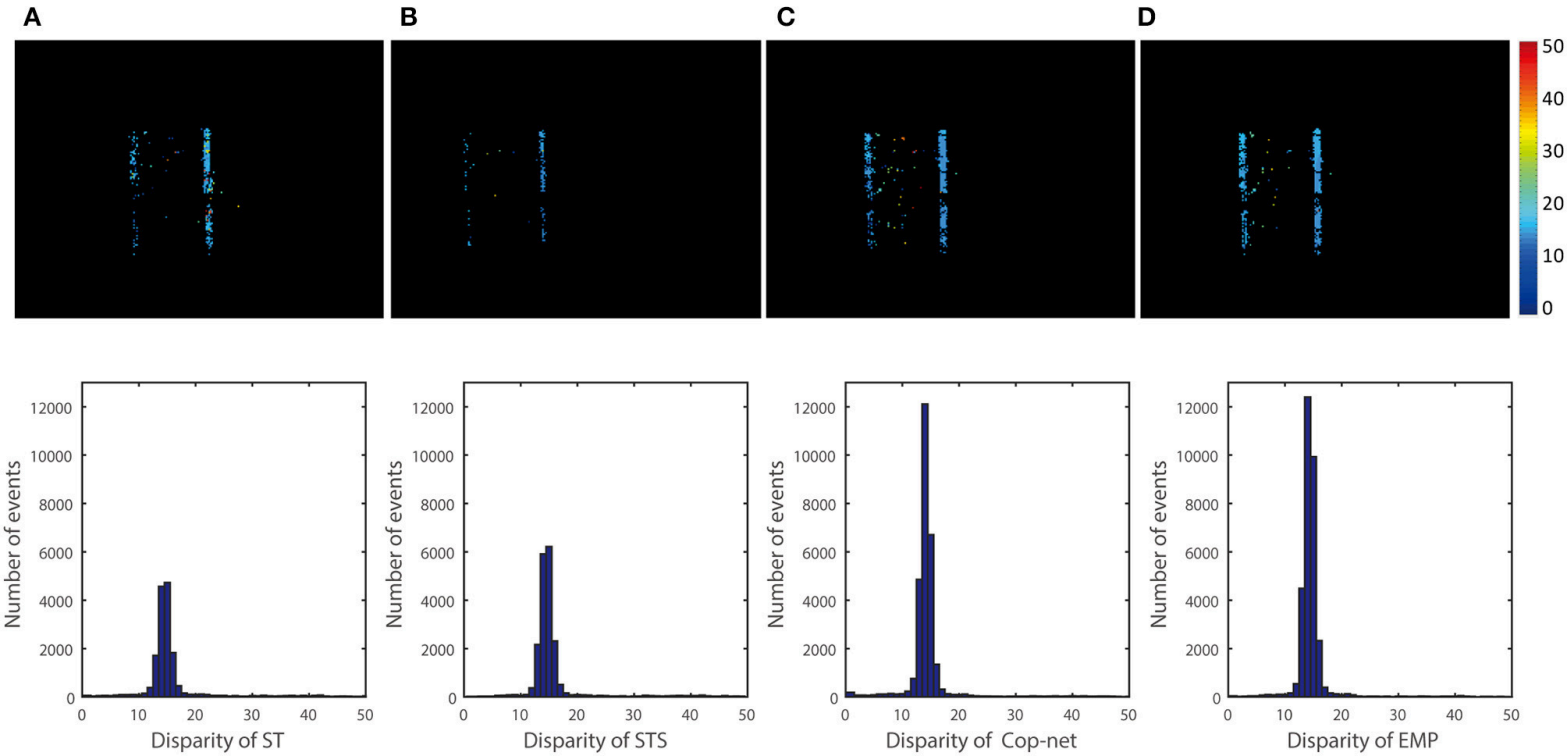
Qualitative and quantitative results from the first scene (One box). The upper row is a color-coded disparity map generated by accumulating 40 ms of disparity estimates. The box is at depth 2 m and the ground truth disparity is 15 pixels, the lower row shows the events disparity histograms over a period of 3 s. From the left to right, the result is extracted by **(A)** ST, **(B)** STS, **(C)** cooperative network, and **(D)** our method.

Figure 6 shows the results for the scene of One box recording using the event-driven disparity output method discussed in section 2.2.4. Due to the method used, only a single disparity estimate may be generated per input event, which is important for fair comparison to other methods using the *estimation rate* metric (on the other hand, disparities output in response to a query can generate more disparity estimates than there are input events).

The top row of Figure 6 shows a snapshot of 40 ms of depth data computed using the ST, STS, Cop-Net, and EMP methods respectively (from left to right). Black grid lines in the disparity map are a side effect of rectification, since some rectified pixel locations may not map to any pixels in the original input event stream.

The bottom row of Figure 6 shows the distribution of disparities computed using each method. The correct disparities lie in the range from 14 to 16. We see that the STS method of Lagorce et al. (2017) has more estimates in the correct disparity range than the ST method. Similarly, the Cooperative Network (Cop-net) approach has more estimates at the correct disparities than both the ST and STS methods. Our EMP method achieves the most disparity estimates within the correct disparity range.

The histograms give a good indication of the distribution of disparities estimated, but do not necessarily indicate that the correct disparity was measured for each event. For example, swapping the disparities associated with two events would result in the exact same histogram, even though both disparities are now incorrect. A more accurate measure is to evaluate the accuracy of each disparity event individually using the metrics described in section 2.4.

Table 1 shows the *estimation rate* and *estimation accuracy* for each method. The Cop-Net and EMP methods clearly provide far more estimates than the ST and STS methods for the one box recording. Of the disparity estimates generated, a higher percentage of the estimates are correct with the EMP method.

**Table 1:** Quantitative results of computation time, the estimation rate, and the estimation accuracy.

**Dataset**	**Method**	**Time (ms/event)**	**Estimation rate (%)**	**Estimation accuracy (%)**
One box	ST	0.023	40.74	68.16
	STS	0.58	49.16	73.33
	Cop-Net	0.75	71.78	75.29
	EMP	1.20	82.16	77.15
One person	ST	0.1	45.43	52.33
	STS	3.6	47.87	56.53
	Cop-Net	1.1	50.77	74.10
	EMP	1.7	94.55	92.00
Two boxes	ST	0.017	34.98	54.25
	STS	0.90	34.34	62.61
	Cop-Net	0.65	61.13	75.29
	EMP	1.2	73.64	82.21
Two people	ST	0.14	43.06	42.59
	STS	6.9	40.89	47.28
	Cop-Net	1.1	49.73	67.08
	EMP	2	92.71	70.64
One person different depth	ST	0.035	37.86	41.33
	STS	0.69	35.42	46.08
	Cop-Net	0.45	53.84	40.78
	EMP	1.01	58.36	61.14

**Figure 7:**
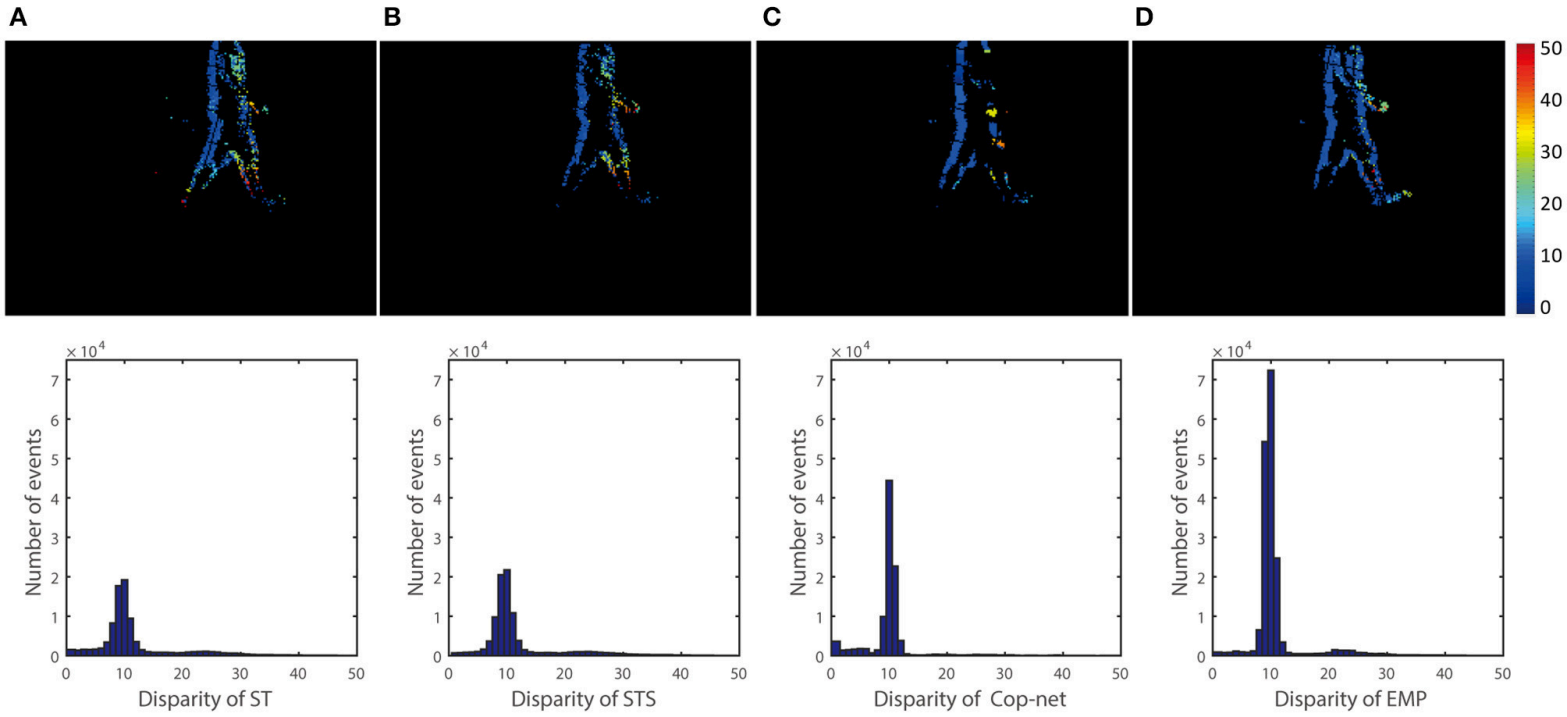
Qualitative and quantitative results of the second scene (One person). The upper row is a color-coded disparity map generated by accumulating 40 ms of disparity estimates. The depth of the person is 3 m and the ground truth disparity is 15. From the left to right, the result is extracted **(A)** ST, **(B)** STS, **(C)** cooperative network, and **(D)** our method.

**Figure 8:**
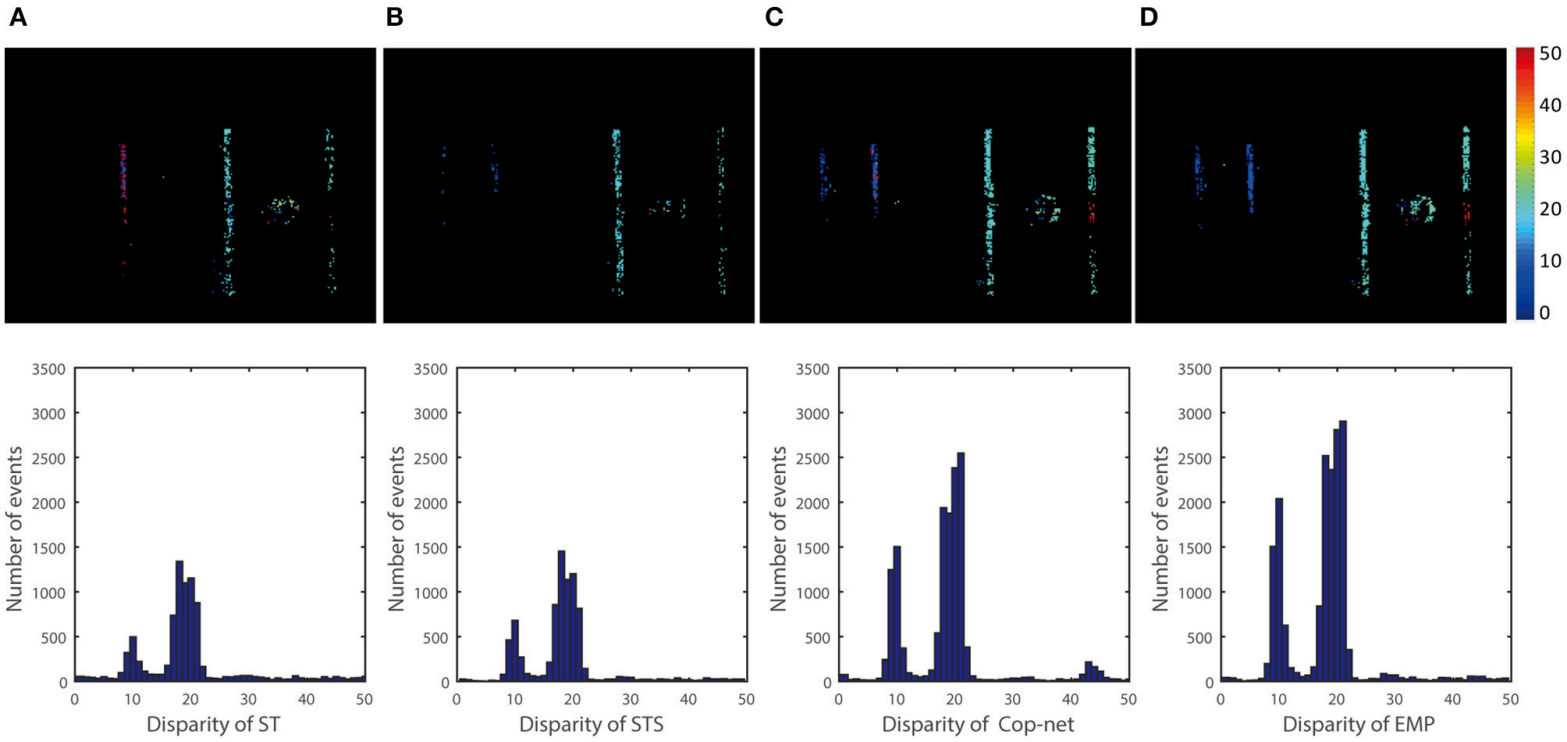
Qualitative and quantitative results of the third scene (Two boxes). The upper row is a color-coded disparity map of a 40 ms-long stream of events for two moving boxes (one is at 1.5 m and another at 3 m). From the left to right, the result is extracted by **(A)** ST, **(B)** STS, **(C)** cooperative network, and **(D)** our method.

**Figure 9:**
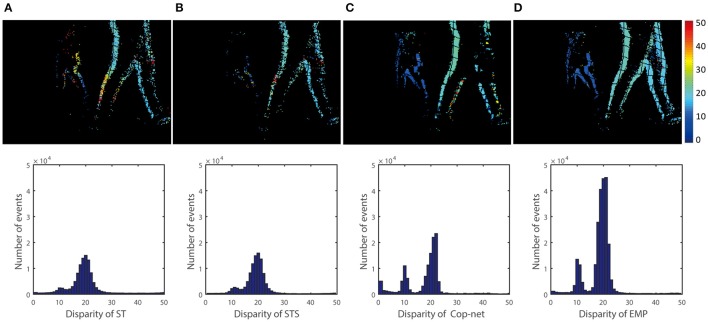
Qualitative and quantitative results of the fourth scene (Two people). The upper row is a color-coded disparity frame map of a 20 ms-long stream of events for two walking people (one at 1.5 m and another at 3 m), the lower ones are events disparity histogram within time of 5 s. From the left to right, the result is extracted by **(A)** ST, **(B)** STS, **(C)** cooperative network, and **(D)** our method.

**Figure 10:**
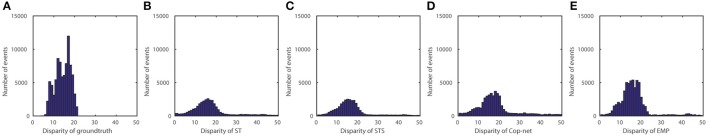
Results of the fifth scene (One person different depth). The events disparity histogram within time of 5s. From the left to right, the result is extracted by **(A)** Ground Truth, **(B)** ST, **(C)** STS, **(D)** cooperative network, and **(E)** our method.

Similar comparisons hold true for the other recordings. Figure 6 shows a simple test case with one box at a constant depth, Figure 8 extends Figure 6 to show that the algorithm can simultaneously detect the depth of multiple objects. Figure 7 extends Figure 8 to shows performance on non-rigid objects and Figure 10 shows the performance when occlusion is present. Figure 10 shows how the algorithm performs when the depth of an object is changing in the scene (for Figures 6–9) each object has roughly constant depth during the recording).

**Figure 11:**
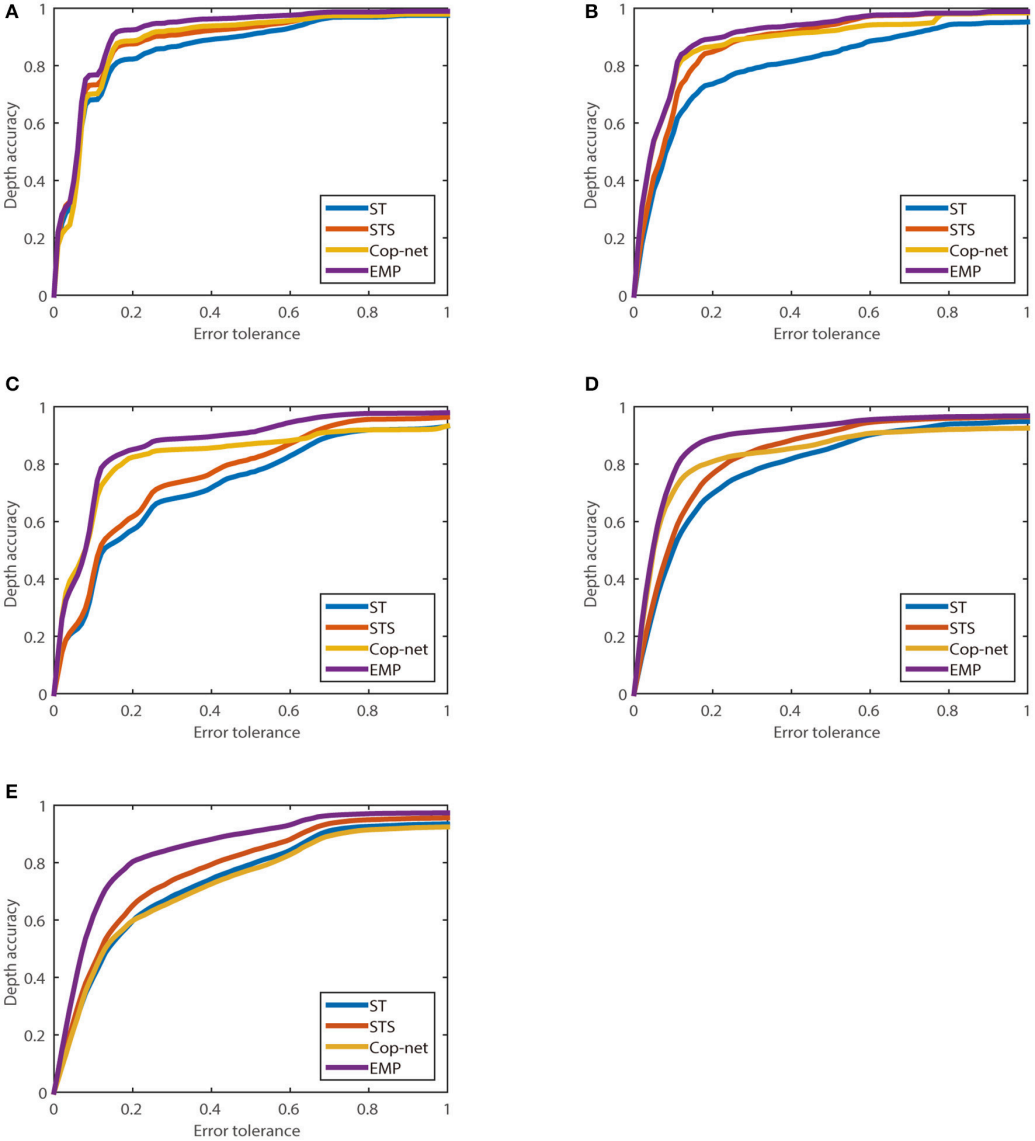
The relationship between depth accuracy *z*_*acc*_(Θ) and error tolerance (Θ) calculated using Equation (8). **(A)** One box, **(B)** Two boxes, **(C)** One person, **(D)** Two people, and **(E)** One person different depth.

Figure 11 shows the percentage of depth estimates, *z*_*acc*_(Θ) (vertical axis), which lie within an acceptable error tolerance, Θ (horizontal axis), as described in Equation (8), for each of the five recordings used.

### 3.2. Map obtained from a query

**Figure 12:**
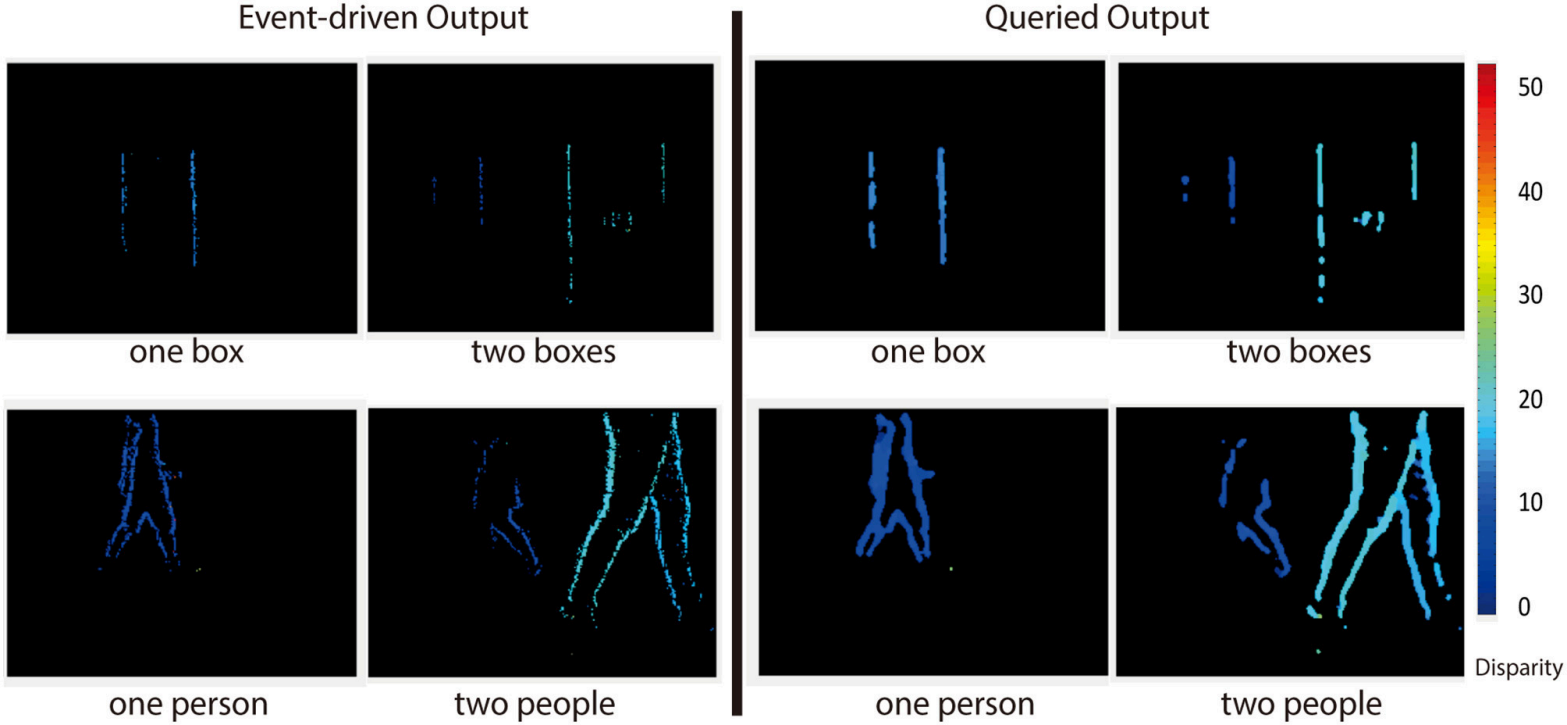
Event-driven (left) vs. queried output (right) for four of the recordings used. A disparity output can be generated at any time by querying the network, and the resulting output is slightly more dense than the event-driven output.

Figure 12 shows the output of the network when queried (see section 2.2.4) vs. accumulating the event-driven output. The result returned from the query is slightly denser than simply accumulating disparity events. This is because disparity estimates can affect neighboring pixels. These neighboring pixels may then report a disparity based purely on disparity information received from neighoring pixels.

This is especially apparent from the fact that the black grid lines are not present in the queried depth map. Even pixels at locations which receive no events (black grid lines) report depth estimates when queried. Beliefs Equation (6) need only be calculated when an output is desired since the state of the network is uniquely described by the messages.

### 3.3. Comparison with frame-based methods

**Figure 13:**
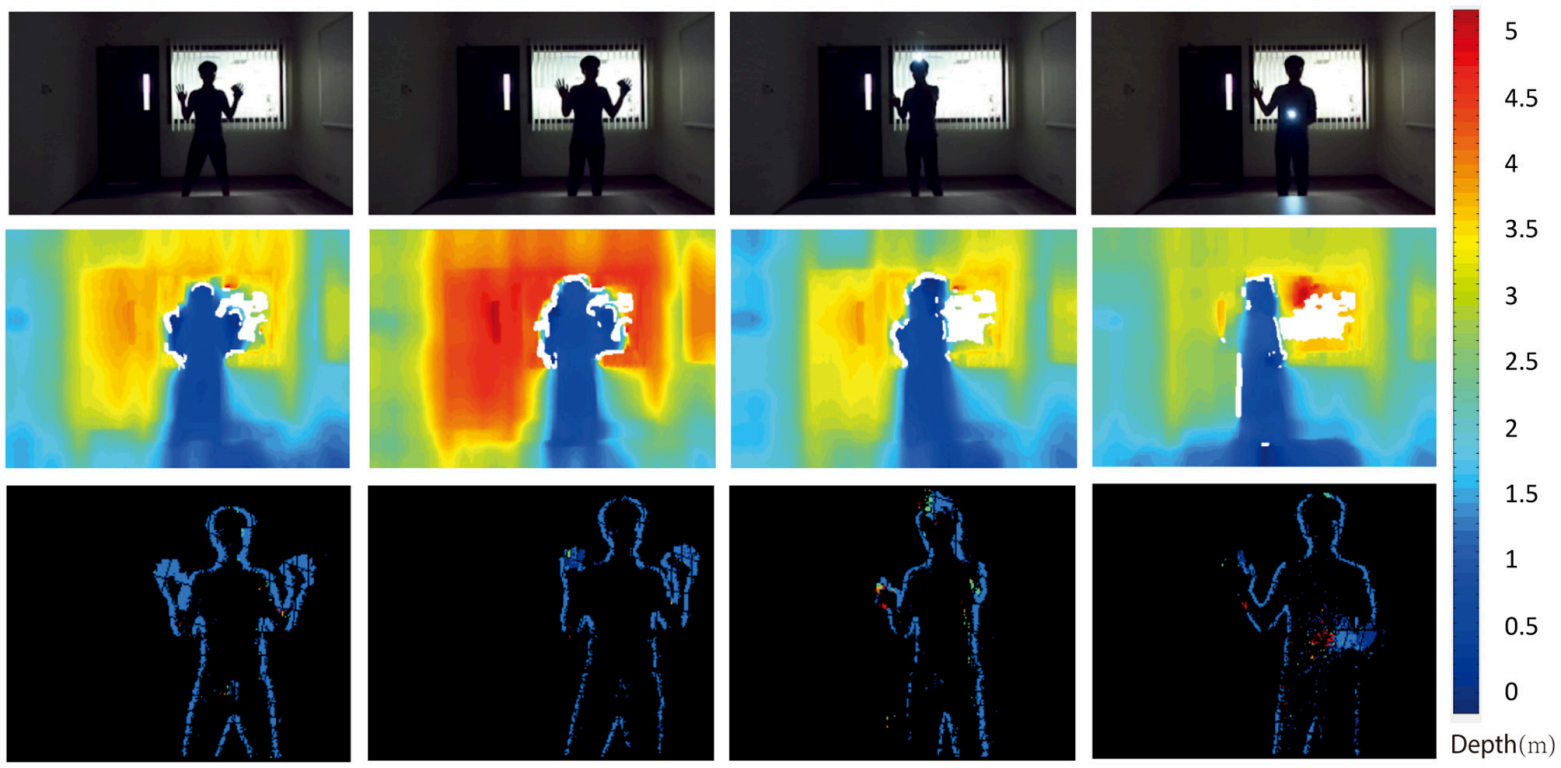
Event-based algorithm vs Frame-based algorithm. The top row show RGB images captured by the ZED cameras. The middle row shows the depth map calculated with the ZED. The bottom row shows the depth map estimated with our EMP algorithm.

Figure 13 shows a comparison between the passive frame-based ZED sensor and the event-based stereo algorithm (EMP) under challenging lighting conditions. The sensors remain static in a high dynamic range scene. The raw images captured by the ZED are shown in the top row. Depth maps generated by the ZED are shown in the middle row. The depth maps are almost fully dense, but do include some missing portions. The depth readings from the ZED are not necessarily constant, even for static regions in the scene. In the second column from the left, the depth measurements are suddenly larger (further) than for the other frames. The outlines of objects (in this case the person) are heavily blurred and the depth discontinuities are not clearly visible.

The event-based sensors can easily handle high dynamic range scenes, as seen in the rightmost image where the depth of the dark arm in from of the dark body is still correctly detected. The output of the event-based algorithm is very sparse, definitely much sparser than the ZED output. The outlines of the person are clearly detected though, suggesting that the event-based depth may play a complementary role to frame based stereo depth detection.

## 4. Discussion

For all of the recordings shown in this paper, the EMP algorithm produces more disparity estimates than the ST, STS, or Cop-net algorithms. Furthermore, when normalizing by the number of disparity estimates generated, EMP still produces on average higher accuracy estimates than the other algorithms. In cases where the ST and STS methods are not able to find a good enough stereo match to generate a disparity output, the Cop-net and EMP approaches can incorporate disparity information from nearby pixels to increase confidence (decrease cost) associated with a disparity output. This results in more output disparities for Cop-Net and ST, as seen in Table 1 and Figures 6–11.

Not only do the Cop-net and EMP algorithms produce more depth estimates (which could also be achieved by simply increasing the allowable cost threshold τ_*o*_), but the estimates produced are more accurate. However, there is a trade-off encountered with the EMP algorithm, since the estimation rate and estimation accuracy comes at the cost of greater computation time compared to the other methods (Table 1). We note here that all tests were run on a CPU, but the Cop-Net and EMP algorithms may allow acceleration on GPUs. In Table 1, the STS algorithm has the longest computation time in recordings of One Person and Two People. One reason is that the Timesurface is estimated in a spatio-temporal region. The more events in the spatio-temporal region, the more time-consuming it is. The One Person and Two People recordings are much more complex than the boxes ones. Another reason is the cost Time is the total cost time divided by the number of detected stereo matches. Our EMP has higher estimation rate which means a large number of detected stereo matches.

For the first four tests shown, objects do not change in depth during the sequence (although different objects may have different depths). To show that the EMP accuracy is not due to the algorithm being biased toward generating outputs at depths which match the objects', a sequence of a person walking away from the sensor was included. The estimation rate and estimation accuracy for this sequence is the lowest of all sequences for all stereo methods presented. Our method has memory of the state (depth) for each pixel. In the case where an object is changing in depth during the recording, the state remembered by the network becomes outdated and incorrect. To properly model the world, we would need to measure the 3D velocity of the camera and objects in the scene, and update the depth map accordingly, but this is beyond the scope of this current work.

The ST and STS methods have no memory of the depths present in the scene (the most recent timestamp for each pixel is remembered, but not the depth). Therefore these methods cannot enforce 3-dimensional constraints which may be present in the scene, such as depth (or disparity) smoothness. The Cop-net and EMP algorithms enforce disparity uniqueness and smoothness, which results in better accuracy for all the static sensor sequences shown.

The EMP method also allows for the current estimate of disparity to be read out at any time by querying the network. The output gives a slightly denser result since it allows pixels with no input events to generate disparity estimates based on the disparities of the nearby pixels. Extensions of this approach may allow for more dense estimation of depth, but this investigation is beyond the scope of this paper.

Event-based depth estimation from stereo still has a way to go if it is to compare favorably to frame-based stereo in terms of spatial resolution and depth resolution because the event-based vision sensors are low resolution compared to their frame-based counterparts. However, event-based stereo can play a complementary role to frame-based stereo, particularly in handling high dynamic range scenes, and estimating the disparity at depth discontinuities where sensors such as the ZED struggle.

We have proposed an event-based stereo depth estimation algorithm which relies on message passing and compared it to previous algorithms on five different recordings. Compared to previous methods, our EMP algorithm produces more estimates, and more accurate estimate, at the cost of a higher computation time per event.

## Author contributions

ZX: Main contributor. Formalized the theory, implemented the experiments and evaluated the results. SC: Co-supervisor. GO: thesis director and main instigator of the work.

### Conflict of interest statement

The authors declare that the research was conducted in the absence of any commercial or financial relationships that could be construed as a potential conflict of interest.

## Footnotes

^1^https://youtu.be/ngJpY1lcbdw

^2^https://github.com/harryxz/EMP (tag: Frontiers)

^3^http://www.ros.org/
